# Sinus arrest following acute lateral medullary infarction

**DOI:** 10.1007/s10072-022-06306-2

**Published:** 2022-08-04

**Authors:** Taha K. Alloush, Adel T. Alloush, Mohammed Sami, Hossam M. Shokri

**Affiliations:** 1 Department of Neurology and Psychiatry, Faculty of Medicine, Ain Shams University, Cairo, Egypt; 2 Department of Geriatrics and Gerontology, Faculty of Medicine, Ain Shams University, Cairo, Egypt; 3 Department of Cardiology, Faculty of Medicine, Al-Azhar University, Cairo, Egypt

**Keywords:** Medullary strokes, Autonomic dysregulation–associated clinical manifestations, Posterior inferior cerebellar artery, Nucleus tractus solitarius, Dorsal vagal nucleus, Permanent pacemaker placement

## Abstract

Lateral medullary syndrome (LMS) is an ischemic stroke of the medulla oblongata that involves the territory of the posterior inferior cerebellar artery. LMS is often missed as the cause of autonomic dysregulation in patients with recent brain stem stroke. Due to the location of the nucleus tractus solitarius (NTS), the dorsal vagal nucleus, and the nucleus ambiguous in the lateral medulla oblongata, patients with LMS occasionally have autonomic dysregulation–associated clinical manifestations. We report a case of LMS-associated autonomic dysregulation. The case presented by recurrent syncope, requiring permanent pacemaker placement. This case shows the importance of recognizing LMS as a potential cause of life-threatening arrhythmias, heart block, and symptomatic bradycardia. Extended cardiac monitoring should be considered for patients with medullary strokes.

## Introduction

Lateral medullary syndrome (LMS), Wallenberg syndrome, or posterior inferior cerebellar artery syndrome results from an infarction in the lateral part of the medulla oblongata. The arteries commonly involved in LMS are the posterior inferior cerebellar artery or the vertebral artery [1] . The lateral medulla contains the inferior cerebellar peduncle, the descending spinal tract, the trigeminal nerve nucleus, vagus nerve and glossopharyngeal nuclei and fibers, spinothalamic tract fibers, and vestibular nuclei [2]. For cardiac autonomic control, the medulla contains key structures involved in both sympathetic and parasympathetic outflow, including the nucleus tractus solitarius (NTS), the dorsal vagal nucleus, the nucleus ambiguous, and the intermediate reticular zone [3].

The risk factors for LMS occurrence are the same as for other types of ischemic stroke: hypertension, smoking, diabetes, and atrial fibrillation [4]. The common pathological mechanisms for lateral medullary infarction are atherosclerosis, arterial dissection, cardiogenic emboli, and small vessel disease in decreasing frequency of occurrence [5] [6].

Lateral medullary syndrome typically has varied neurologic manifestations. LMS is clinically characterized by ipsilateral Horner’s syndrome, ipsilateral ataxia, contralateral hypoalgesia of the body, and ipsilateral facial hypoalgesia [2] [7]. While sensorimotor dysfunction presents as a predicted pattern of clinical signs and symptoms, autonomic dysfunction is usually less clinically apparent and can be easily mistaken as a concomitant pathology in the end organ it affects. Due to the location of the baroreceptor regulatory center in the lateral medulla oblongata, patients with Wallenberg’s syndrome occasionally have autonomic dysregulation [2]. Infarction of this intricate territory can lead to lability of vascular tone and heart rate, resulting in recurrent syncope [8] [9] [10].

Medullary infarction can also cause sudden cardiorespiratory arrest, but this is uncommon [9]. The causes of sudden death following medullary infarction include sudden cardiac arrest, respiratory arrest, and arrhythmia [11] [12] [13]. These events can even occur in patients with stable hemodynamic and neurological conditions [14] [15] [16]. Cardiac arrhythmia is one of the mechanisms by which cardiorespiratory arrest occurs [16]. Medullary lesions can cause autonomic instability which can precipitate fatality. It is expected that ischemic lesions of the solitary tract nuclei, associated with some lateral medullary infarctions, could lead to cardiorespiratory arrest [17] [18]. In this case, we present a case of an unusual pattern of cardiac arrhythmia 10 days after LMS, caused by autonomic instability following infarction of the vagus nerve nuclei in the medulla.

## Case report

A 64-year-old chronic smoker man presented with vertigo, vomiting, hiccough, dysphagia, hoarseness of voice, unsteadiness, and falling towards the left, associated with left-sided headache of 6-h duration. He had a past history of hypertension and type II diabetes.

On examination, his blood pressure was 140/90 and his pulse was regular at 66 beats/min on admission. The neurological examination revealed left-sided miosis associated with narrowing of the palpebral rima and horizontal nystagmus, most apparent on left lateral gaze. He had dysphonia, impaired palatal elevation, and difficulty swallowing with nasal regurgitation. There was dysmetria on the left side, with severe truncal ataxia and a tendency to fall towards the left side. Sensory examination revealed diminished pinprick sensation on the left side of his face and the right side of his body. Cardiovascular, respiratory, and abdominal examinations were all unremarkable.

**Fig. 1:**
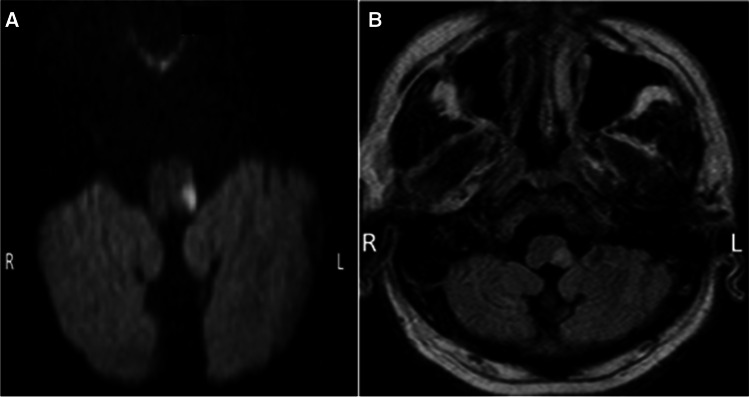
MRI brain, **A** axial DWI, and **B** FLAIR show an acute left-sided dorsal lateral medullary infarct

An MRI brain revealed restricted diffusion, denoting acute infarction in the left lateral medulla oblongata and a hyperintense lesion on FLAIR imaging denoting acute-subacute infarction (Fig. 1).

Routine laboratory investigations and baseline ECG were normal. Transthoracic echocardiogram (ECHO) revealed a non-dilated left ventricle, with moderate wall thickness, normal systolic function, and no evidence of intracardiac thrombi or masses. Carotid and vertebral arteries ultrasound revealed mild diffuse atherosclerotic changes with no significant stenosis.

The patient presented outside the recommended window for thrombolytic therapy. As a result, he was treated with a high-intensity statin, aspirin, aggressive blood pressure, and blood sugar control in accordance with subacute stroke treatment guidelines. He made a good recovery with improvement of his neurological symptoms. Within 4 days, he improved dramatically. His speech returned to normal, his dysmetria resolved, and he was able to sit unassisted and stand with mild support. No arrhythmias were identified during cardiac monitoring. The patient’s dysphagia persisted, requiring placement of a nasogastric tube. After 1 week since admission, the patient started to eat soft meals and he was discharged from the hospital with home physical therapy visits.

Two days after discharge, the patient experienced multiple unprovoked syncopal episodes. The syncopal episodes were brief and were predominately associated with pallor, open eyes, with no tongue biting, incontinence, vertigo, nausea, or vomiting. The condition was associated with immediate and complete recovery. No seizure-like activity or confusion was noted and both the patient and his caregiver denied any history of similar episodes of syncope prior to this admission.

During the patient’s hospital stay, another witnessed syncope occurred, during which bradycardia was observed on the patient screening monitor, and the patient was investigated with 24-h Holter ECG to document and diagnose the type of bradyarrhythmia observed on the monitor.

**Fig. 2:**
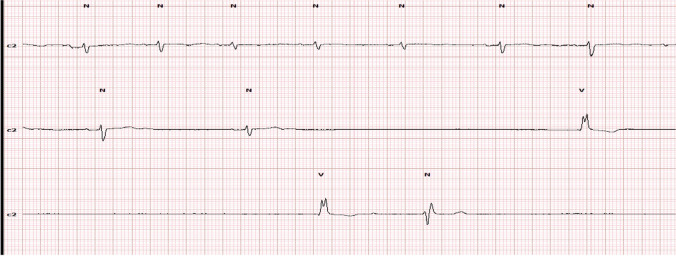
Long strip showing sinus arrest with idioventricular escape rhythm @heart rate 13 bpm @ 1:28 PM

**Fig. 3:**
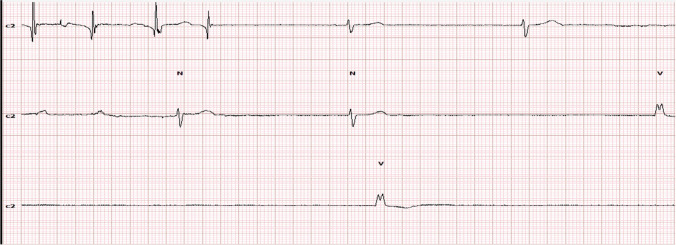
Onset of bradyarrhythmia sinus arrest with junctional rhythm followed by ventricular escape rhythm

Furthermore, the Holter showed that minimal HR was 16 bpm during waking hours (idioventricular escape rhythm). It was initiated by sinus arrest with junctional escape rhythm at a heart rate of 40 bpm, followed by junctional arrest and idioventricular escape rhythm (Figs. 2, 3). The longest RR interval was 4.6 s, which was associated with syncope. The maximal HR was 128 bpm (sinus tachycardia that showed gradual onset and termination) with a normal AV interval along with recording hours. Regarding heart rate variability, the rMSSD was 78. Coronary angiography was done to exclude ischemic sinus pauses and showed no significant stenosis of epicardial coronary arteries.

Permanent pacing was performed using Dual chamber pacemaker (ENDURITY MRI ST JUDE) programmed on DDDR mode with basal rate 60 bpm, sensed AV delay 180 ms, and paced AV delay 200 ms with VIP 50 ms, and rate drop response was programmed to pace at HR 70 bpm.

The syncopal attack totally improved and the patient was discharged.

Six weeks after implantation, interrogation of the pacemaker showed that pacing accounted for 5.6% of total beats. No sustained atrial or ventricular arrhythmias were recorded.

## Discussion

A 64-year-old male patient with classic left LMS. The patient reported no past history of cardiac problems, TIA, or stroke. Also, he had a normal ECG at presentation and a normal ECHO. Whether the stroke was cardioembolic or atherosclerotic, the negative history and initial cardiac workup suggest the second one.

Importantly, the patient developed recurrent syncopal attacks, despite good motor improvement with appropriate medical therapy. The syncopal episodes most probably were secondary to autonomic dysregulation from lateral medullary infarction. Fortunately, the syncopal episodes responded to permanent cardiac pacing, supporting the hypothesis that syncope was predominantly the result of sinus arrest (cardioinhibitory response), as opposed to dysautonomia-associated hypotension (vasodepressor response), for which the role of cardiac pacing is poorly defined [10]. Sudden cardiorespiratory arrest can occur several days after lateral medullary infarction especially when the patients are stable medically and neurologically. The extension of the lesion, possibly the ischemic penumbra, may affect the brain stem cardiac and respiratory centers together with autonomic pathways [19]. The patient had no prior history of stroke and his MRI showed no areas of old infarctions. In particular, insular and hypothalamic regions appeared to be free of acute and previous strokes on MRI. Those areas are especially known to be associated with autonomic dysregulation [20] [21].

This suggests a different localization of the stroke-mediated autonomic dysregulation in the present case, specifically the medulla. The medulla contains key structures involved in autonomic regulation, including the dorsal vagal nucleus and the nucleus tractus solitarius. LMS may cause acute infarction of these two nuclei or their pathways, resulting in alteration of parasympathetic and sympathetic outflow to the sinoatrial and atrioventricular nodes, with potentially life-threatening effects [22]. Similar case reports showed sudden cardiac arrest, respiratory arrest, and sinus bradycardia or sinus pauses following acute medullary infarction [11] [12] [13] [14] [15]. Moreover, the resolution of recurrent syncopal attacks after pacemaker placement suggests symptomatic benefit, which may be applicable in similar selected cases.

**Fig. 4:**
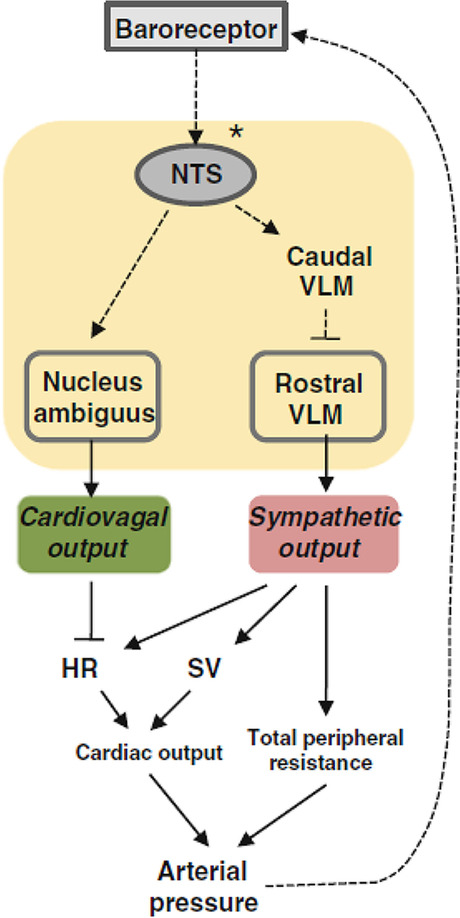
Schematic diagram of the baroreceptor reflex. This reflex involves connections between the nucleus tractus solitarius (NTS) and the ventral medulla and constitutes a feedback mechanism (adapted from Hong et al. [9])

Stimulation of the NTS leads to inhibition of neurons in the rostral ventrolateral medulla (RVLM), resulting in a decrease in the sympathetic outflow (Fig. 4) which leads to bradycardia and low blood pressure [23].

Electrolytic lesions in the commissural NTS prevent baroreceptor-mediated tachycardia but not bradycardia, whereas lesions in the subpostremal portion of the NTS prevent baroreceptor-mediated bradycardia but not tachycardia [24]. In our case, we hypothesize that there was sympathetic dysfunction and unopposed parasympathetic activity that was supported by high rMSSD in Holter monitoring.

Reversibility of these cases is still unknown due to the rarity of reported cases. One of the reported cases showed complete recovery 4 days after the event and only temporary pacing was required [9]. Other reported cases showed persisted bradyarrhythmias for a longer time requiring permanent pacemaker implantation [10]. In our case, the interrogation of the device showed that the patient was still in need for pacing even 6 weeks after device implantation.

## Conclusions

Although lateral medullary syndrome has often a good functional recovery, in some cases, it is a potential cause for life-threatening arrhythmias, heart block, and symptomatic bradycardia. In-hospital Holter monitoring is recommended following LMS stroke. Pacing either temporary or permanent may be necessary, in order to control sinus arrest secondary to dysautonomia.
